# Control of Cattle Ticks and Tick-Borne Diseases by Acaricide in Southern Province of Zambia: A Retrospective Evaluation of Animal Health Measures According to Current One Health Concepts

**DOI:** 10.3389/fpubh.2018.00045

**Published:** 2018-03-27

**Authors:** Gabrielle Laing, Maurizio Aragrande, Massimo Canali, Sara Savic, Daniele De Meneghi

**Affiliations:** ^1^Department of Epidemiology and Population Health, University of Liverpool, Liverpool, United Kingdom; ^2^Department of Agricultural and Food Sciences, University of Bologna, Bologna, Italy; ^3^Scientific Veterinary Institute ”Novi Sad”, Novi Sad, Serbia; ^4^Department of Veterinary Science, University of Turin, Grugliasco-Torino, Italy

**Keywords:** public health, environmental impact, economic evaluation, OH index, one health

## Abstract

One health thinking for health interventions is increasingly being used to capture previously unseen stakeholders and impacts across people, animals, and the environment. The Network for One Health Evaluation (NEOH) proposes a systems-based framework to quantitatively assess integration and highlight the added value (theory of change) that this approach will bring to a project. This case study will retrospectively evaluate the pioneering use of a One Health (OH) approach during an international collaboration (satellite project to tackle production losses due to tick-borne disease in cattle in Southern Zambia in late 1980s). The objective of the evaluation is twofold: retrospective evaluation the OH-ness of the satellite project and identification of costs and benefits. Data for evaluation was recovered from publications, project documents, and witness interviews. A mixed qualitative and quantitative evaluation was undertaken. In this case study, a transdisciplinary approach allowed for the identification of a serious public health risk arising from the unexpected reuse of chemical containers by the local public against advice. Should this pioneering project not have been completed then it is assumed this behavior could have had a large impact on public wellbeing and ultimately reduced regional productivity and compromised welfare. From the economic evaluation, the costs of implementing this OH approach, helping to avoid harm, were small in comparison to overall project costs. The overall OH Index was 0.34. The satellite project demonstrated good OH operations by managing to incorporate the input across multiple dimensions but was slightly weaker on OH infrastructures (OH Ratio = 1.20). These quantitative results can be used in the initial validation and benchmarking of this novel framework. Limitations of the evaluation were mainly a lack of data due to the length of time since project completion and a lack of formal monitoring of program impact. In future health strategy development and execution, routine monitoring and evaluation from an OH perspective (by utilizing the framework proposed by NEOH), could prove valuable or used as a tool for retrospective evaluation of existing policies.

## Introduction

Within global health, there is a move toward more integrated planning and delivery approaches that can yield more efficient, effective, and equal outcomes than traditionally siloed approaches to health challenges. The use of One Health thinking to identify stakeholders and capture impacts across people, plants, animals, and the environment has the potential to avoid harm and identify benefits otherwise unseen. The Network for One Health Evaluation (NEOH) proposes a novel evidence-based framework to quantitatively assess integration and highlight the unique benefits (theory of change) that this approach will bring to a project (1, 2).

In 1987, Italian development and research institutions (Ministry of Foreign Affairs, Development Cooperation Office and the *Istituto Superiore di Sanità*, Roma: ISS, Rome) responded to an emergency call of the Zambian government (Ministry of Agriculture, Department of Veterinary Services) for help to control deaths and production losses in cattle caused by *Theileria parva* infection, also known as Malignant Theileriosis, transmitted by *Rhipicephalus appendiculatus* ticks. This Animal Health Programme (AHP) took place in the Southern Province of the country and continued until 1992, but here we concentrate on a 2-year (1988–1989) satellite project (One Health Initiative, OHI). The OHI assessed the implications of the AHP for other stakeholders and implemented public health education and risk mitigation activities in support of the main project. The information and data reported in this paper, concerning the activities of the AHP and OHI are based on the following publications and project reports by Ghirotti et al. (3, 4), De Meneghi et al. (5–7), Camoni et al. (8), Scorziello et al. (9), and to which reference will be made throughout the text.

### The Animal Health Programme

**Figure 1:**
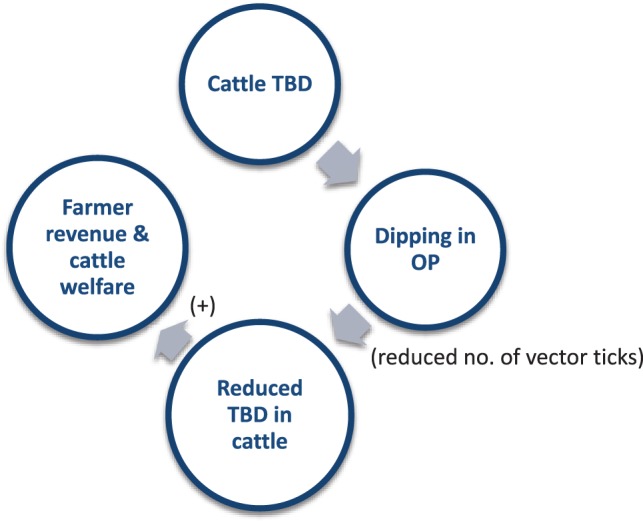
Flow of operations relating to the Animal Health Programme (AHP).

The expected outcome of the AHP was to reduce tick-borne diseases (TBD) in cattle, in particular Theileriosis, to avoid losses and increase cattle productivity. The expected impact was to increase animal welfare and stabilize or increase farmers’ income. The AHP was conceived as a typical animal health intervention meaning that animal health was the focus of the emergency project with no other initial considerations made for other dimensions of the intervention (Figure 1).

The AHP required farmers to attend communal dipping tanks (DTs) with their cattle on a weekly basis during peak tick season (i.e., November–May, 7 months), according to a strategic dipping regime. Approximately 530,000 cattle (45% of the national herd) attended 130 communal dip-tanks in Southern Province over the study period. Cattle would approach the dip-tank in single file along a race and make a small jump into the tank so that head and body would be briefly but completely submerged in an acaricide solution. Cattle would exit into a draining area where excess fluid would drain back into the tank. The dipping liquid contained an organophosphate (OP; in this case, chlorphenfinvos) as the active ingredient against ticks. The dipping process thus required transport, stocking, and handling of OP by dip-tank operators (DTOs). Stocks of concentrated dip fluid were stored in a main central storehouse, and in small buildings at each Veterinary District Office in Southern Province, and transported in 5 l canisters from district storage points to the dip-tanks. Often this was by hand, bicycle, or motorbike. In order to prepare the fluid for the dip-tank, concentrated fluid from the canister had to be diluted with water according to the manufacturer’s instructions. At the end of each season the dip-tanks were emptied of fluid (and occasionally this was also performed during the season if the tank became too contaminated). Existing disposal procedures were to discard fluid directly onto fallow land or *via* a decantation pit (where available) to allow degradation of active ingredients. Operation and maintenance of dip-tanks, preparation of dip-tank fluid, and provision of DTOs fell under the responsibility of the Zambian authorities. All staffing, testing, and transport costs associated with the satellite project were covered by Italian sources. Farmers paid a small fee toward cost of treating their cattle on a cost-recovery basis (approximately Zambian Kwacha 50 cents per dip, 0.04 USD in 1988).

### The Acaricide Active Ingredient and Potential Health Risks

The OP based acaricide used in the AHP was Steladone 300 EC (Ciba-Geigy) and provided by the Italian Cooperation Programme, who also offered technical assistance. OPs have been successfully used to control vectors of animal disease and plant pests elsewhere. The active ingredient used, Chlorfenvinphos, acts as a cholinesterase inhibitor in ticks but this also occurs in humans, affecting both the peripheral and central nervous system. Toxicity may occur following direct contact, ingestion, or inhalation of fumes (10). The use of this and other OPs in developing countries worldwide had been associated with 3 million acute poisoning cases per year (at the time of the original study), including 220,000 yearly fatalities (11). Risks associated with long-term exposure to small doses are also expected to have some human health impacts but there is little existing literature and risks are largely un-quantified in developing nations (12).

Residues of OPs are expected in soils, water, and animal products such as milk, and maximum limits for residues have been set by the Codex Alimentarius (13). Signs of acute toxicity can range from mild to severe. Severe intoxication can result in generalized convulsions and death through respiratory or cardiac failure but mild intoxication may be difficult to diagnose. Signs of mild intoxication include nausea, headache, miosis, vomiting, weakness, and giddiness (10).

The highest risk of toxicity occurred when staff were handling concentrated acaricide fluid or during storage in poorly ventilated facilities. There was also a smaller risk associated with exposure to the diluted dip-tank solution, especially as the dipping process often results in large amounts of displaced fluid around the tank onto surrounding staff or from the risk of staff accidentally falling into the tank. Reducing the risk of intoxication was achieved through limiting direct contact, inhalation or ingestion of concentrated acaricide by the use of personal protective equipment (PPE), and practice of safe operating procedures for DTOs. An antidote (atropine) can be administered for incidents of acute poisoning, and this was distributed by the satellite project (OHI) to the district veterinary offices, local health-care centers, and district hospitals. There were two recorded incidences where people fell into dip-tanks during the study period, but swift action to clean the acaricide off prevented illness (9).

### The Satellite Project (One Health Intervention: OHI)

The implementation of the AHP had effects beyond those directly expected from the dipping of cattle in OPs. As mentioned above, the dipping process requires that fluids used to treat animals be normally disposed onto fallow land at the end of the dipping period, but OPs can persist in soil for up to 30 weeks. Thus, it was assumed the disposal method adopted in the dipping process negatively impacted environmental health and could potentially be a source of contamination of ground water, agriculture, or wildlife. The OHI provided instructions to DTO and stakeholders to implement a decantation pit step in the disposal procedure where toxic substances could degrade before disposal onto fallow land (7, 9).

A second aspect concerned the disposal of OP canisters. The initial agreement with DTOs was to give empty canisters to the district offices. However, local community members were permitted to reuse the canisters to store fuel (i.e., kerosene, petrol, lubricant oil). In spite of recommendations, it was noted that containers were sometimes being used also to store consumables. Toxicology testing showed that even after repeated washing, consumables such as water stored in the canisters, had unsafe residue limits and may be putting the public at risk of acute toxicity. The reuse of these canisters for consumables was an unexpected outcome of the AHP.

Additionally, instructions to farmers not to use milk produced by cattle for at least 12 h after dipping were commonly disregarded. Milk testing showed unsafe residue limits for human consumption until 18–24 h post-dipping and withdrawal times for milk for animal consumption, mainly suckling calves, were only deemed safe 5 h post-dipping (8).

Finally, it was noted by the dipping process managers that people (DTOs and farmers) view the risk to their personal health from exposure to acaricide as low. This was suggested to arise from the perception that the acaricide is a form of “medicine” and that the causal pathway between exposure and illness is not always obvious. Alongside a lack of awareness of the risks from exposure, the (initial) unavailability of PPE was also thought to be a barrier for practice of safe behaviors by DTOs.

Education campaign messages used a variety of multimedia materials, including leaflets, community meetings and radio and TV programs, in particular a radio drama aiming to raise awareness of exposure risks from post-dipping milk consumption and acaricide canister reuse. Means were adapted for use in local, regional, and national education campaigns.

This case study aims to demonstrate how typical intervention strategies for animal health may result in unexpected social impacts due to the insufficient or poor consideration of intervention complexity in the specific social context, e.g., local cultural attitudes to the acaricide and canister reuse, exposing local operators, people, and the environment to increased risk of poisoning and pollution. In this respect, an *ex post* re-consideration of the satellite project may show features of the current OH approach, that we want to evaluate.

The objective of the evaluation is twofold:

(a) retrospective evaluation of the OH-ness of the satellite project
(b) identification of the costs and benefits of the satellite project, and their quantification, where possible with the data available.

This will be a mixed qualitative and quantitative evaluation, based on the reconstruction of the cost of the satellite project and a quantitative consideration of the benefits for the population and the on-site health management system.

The objective of the evaluation can be structured according to the following evaluation questions:

- what is the degree of OH-ness of the satellite project according to the main components of OH outlined in the NEOH framework?
- did the use of an OH approach to an animal health and a veterinary public health intervention prove valuable over a siloed disciplinary approach (theory of change)?
- what were the costs of the satellite project?
- what were the benefits?

## Materials and Methods

### OH-ness Evaluation: Systems Map, One Health Index, and One Health Ratio (OHR)

The evaluation method follows guidelines outlined in the journal’s introductory paper (1). The first aspect for the evaluation was the identification of the system, its dimensions, boundaries, and potentially relevant interacting or independent components. In this case study, the system includes the AHP and satellite project as a subsystem (including their functional links). The system also defines stakeholders as “*any individual, group or organization who may affect, be affected by, or perceive themselves to be affected by a decision or activity*” and then a subgroup of actors “who act or take part” in the system. The dimensions characterize the system and may take place at different scales (e.g., the geographical dimension includes various levels of scale, from local to international). Dimensions also take account of the social context in which they operate (people, society, institutions) and their behavior in the context of OP use (including the related structure, infrastructure, and equipment). The sequence of inputs, outputs, outcomes, and impact pathways were plotted graphically in a logical framework. Outcome mapping was used in building the logical framework, grouping outcomes as disciplinary or interdisciplinary and sequential impacts as first or second order. The contribution of the OHI will be compared to the three pillars of sustainability: society, environment, and economy, such as interspecies equity, human welfare, and efficiency in this example. A theory of change was described from the added value the OHI contributed in the system.

Recovery of data from the satellite project for OH-ness evaluation was through publications, project documents still available, and witness interviews including contributions by one of the coauthors (Daniele De Meneghi) and colleagues formerly working in the AHP. These data were analyzed during the evaluation process in accordance with the recommendations and tools set out in the NEOH handbook.^1^ Briefly, process evaluation was through semi-quantitative assessment under six major themes, split into operational aspects (thinking, planning, working) and infrastructure (learning, sharing, systemic organization). OH thinking explores the dimensions and scales within the system and the context-specific suitability of the OHI in matching these. OH planning assesses resource allocation and appropriateness and adaptability of plans to address the objectives of a transdisciplinary project. OH working focuses on the disciplinary diversity and placement of appropriate leadership and management to promote non-hierarchical relationships and transdisciplinary working within the team and for project outputs. In a similar line, systemic organization looks at the implementation of shared leadership and governance involving all stakeholders and engaging those from all to avoid a silo mentality. Evaluation of learning examines knowledge exchange infrastructure and how this supports learning within the system and in the broader environment. Finally, evaluation of sharing infrastructure looks to reward projects where there is facilitation for good quality data sharing, for example, where data are presented with recognition of potential bias; in a suitable format to allow merging of data from multiple sectors; and with appropriate measures to uphold confidentiality.

Scores were allocated following review of criteria relevant to each theme and compared to a context-specific benchmark as determined by the evaluator as appropriate to the project under review; where full realization of the ideal scenario was worth 1.0. The scores for each theme were plotted onto the spokes of a spider diagram to allow visualization of overall project integration and the balance between operational and infrastructure elements. As outlined in the introductory article by Rüegg at al., a quantitative overall One Health Index was calculated as a ratio of the area enclosed by the points when plotted onto the spider diagram to the area enclosed if all spokes were equal to 1, according to the following formula:

$$\text{OH Index}=\frac{\left[\begin{array}{l} \left(\text{ScP}\times \text{ScT})+(\text{ScL}\times \text{ScP})\text{+}(\text{ScL}\times \text{ScS}\right)\\ \text{ +}(\text{ScO}\times \text{ScS})\text{+}(\text{ScW}\times \text{ScO})\text{+}(\text{ScT}\times \text{ScW}) \end{array}\right]}{6},$$

where ScP is the score obtained in OH planning, ScL is the score obtained in learning infrastructure, ScS is the score from sharing infrastructure, ScO is the score from systemic organization, ScW is the score from OH working, and ScT is the score from OH thinking.

A comparison between the OH operations and infrastructure was made by dividing the area enclosed by the points associated with OH operations by that for the infrastructure, which gives the OHR:

$$\text{OHR=}\frac{\left(\frac{\text{ScO}\times {\text{ScW}}^{\text{2}}}{\text{ScO+ScW}}\right)\text{+}\left(\text{ScW}\times \text{ScT}\right)\text{+}\left(\text{ScT}\times \text{ScP}\right)\text{+}\left(\frac{{\text{ScP}}^{\text{2}}\times \text{ScL}}{\text{ScP+ScL}}\right)}{\left(\frac{\text{ScP}\times {\text{ScL}}^{\text{2}}}{\text{ScP+ScL}}\right)\text{+}\left(\text{ScL}\times \text{ScS}\right)\text{+}\left(\text{ScS}\times \text{ScO}\right)\text{+}\left(\frac{{\text{ScO}}^{\text{2}}\times \text{ScW}}{\text{ScO+ScW}}\right)}.$$

An external evaluator (Gabrielle Laing) consulted with the internal evaluator (Daniele De Meneghi) to compile the information for evaluation with guidance from Maurizio Aragrande and Massimo Canali. Stakeholders Dr. Maria Scorziello Biocca (MD, formerly at Ministry of Health as public health specialist seconded to the ISS Rome) and Dr. Prof. Silvana Diverio (DVM, former grantee of the *Istituto Italo-Africano*, now at the University of Perugia), assisted in data recovery and contributed to OH-ness scoring. Evaluation criteria were described by Gabrielle Laing and scoring for each question reviewed by Daniele De Meneghi, Maurizio Aragrande, and Massimo Canali. A fourth external evaluator (Sara Savic) was asked to review the scoring and criteria based on only the information presented in this paper.

### Economic Evaluation

In order to measure interdisciplinary outcomes and translate evaluation findings in a transdisciplinary way (i.e., between disciplines and societal fields such as the private and public sector), it is desirable to use a common metric. In this instance, an economic evaluation was selected.

According to a consolidated and widely accepted definition, the full economic evaluation of an intervention requires the identification of its costs and outcomes and the comparison with the costs and outcomes of one or more alternative actions (being the situation without any intervention one of the possible alternatives). Partial economic evaluations are performed when one of those conditions is not fulfilled, i.e., only costs or only outcomes can be evaluated, or alternative solutions are not examined [about the distinction between full and partial economic evaluations, see Ref. (14–21)].

For the satellite project, it was only possible to retrieve data on the expenditure of the intervention from the archives of the institutions concerned and from documentation provided by experts who participated in the project planning and implementation at different levels. Available data on the outcomes are incomplete as an *ex post* evaluation was not planned for this OHI, partly because of the emergency nature of the main AHP project and partly because it was not an ordinary practice for this type of project at that time. The time elapsed and the poor follow-up of the project also hindered the identification of such data within the framework of the current evaluation. This makes a comparison with costs and outcomes of alternative actions impossible.

This evaluation, therefore, reports the description of the expenditure and outputs of the satellite project, identifying the benefits to the population of the beneficiary country, based on the information presented and on estimations about acaricide use in the targeted DT stations.

## Results

### Systems Map and Theory of Change

A systems map for the AHP and OHI, based on the elements outlined in the Section “Introduction” and in particular

**Figure 2:**
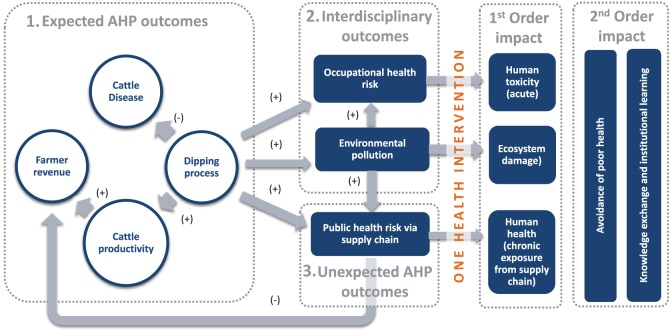
Logical framework showing Impact Pathways describing interactions between inputs, outputs, outcomes, and impact of the system of the Animal Health Programme (AHP) and OHI. The system relating to the primary AHP (Box 1) showing the “theory of change,” i.e., interdisciplinary outcomes arising from the AHP (Box 2) and highlighting the impact pathways on which the One Health Initiative acts with both first order and second order impacts assumed. The figure also shows where unexpected outcomes (Box 3) were captured through the adoption of the transdisciplinary approach used in this case.

– the technological specification of the dipping process and its aim,
– the social and physical environment of the process,
– the objective risks related to OP use,
– the practices actually adopted by the operators and the local population linked to DTs operations and OP handling and related material and facilities (storage facilities, empty canisters, the environment, etc.)

were used to draw the series of consequences (impact pathway) started by the AHP and shown by the logical framework in Figure 2.

The system includes both the AHP and the satellite project as the former involved or induced the latter, in the sense that the existence of the satellite project strictly depends on the implementation of the AHP, its limits, and unexpected consequences. System boundaries are determined by the considerations below:

(i) The fact that the AHP was targeted to a specific region of Zambia (Southern province) by a technical and political decision, exogenously established a geographical limit of the system. This decision formed an initial assumption for the next steps of the system identification process. Together with the dimension of the cattle herds treated (traditional livestock breeding system), these elements allowed for the identification of the main geographical, social, and physical dimensions (boundaries) of the system.
(ii) The dipping process was implemented within these boundaries, allowing for the identification of technological specifications and organization (e.g., the number of risk animals to be treated, the number, dimension, and location of DTs, period and frequency of treatment, dipping routine, use of inputs, etc.). DTs are the technological devices at the core of a process. They imply the use of inputs, the production of outputs, and a flow of actions and consequences (see above AHP process description), and they can be considered basic units of a system characterized by strategy (reducing or eliminating Theileriosis in cattle) and aims (increasing cattle productivity and farmers’ revenue). Starting from these elementary units, risk factors can be analyzed for people directly involved in the dipping process.
(iii) The observation of social behaviors related to the dipping process (as described in Section “Introduction”) allowed for the identification of current and potential risk originating from the AHP but affecting greater dimensions. This expanded the complexity of the system also including other relevant local social units (mainly consumers of milk from treated cows, users of disposed canisters to store food, farmers, and villagers in general) beyond the DTOs, who were identified as the people at highest risk. The observation of the real dipping process development in different treatment sites allowed for the identification of actions, behaviors, and consequences according to a model which can be conceptualized using a systems-based approach.

The satellite project (OHI) is the initiative operating within the system shown in Figure 2 and described in Table 1. The OHI aimed to reduce or eliminate exposure to the risks of OP use, which were interdisciplinary alongside any unexpected impacts of the dipping process in the context of the AHP. The main stakeholders were the DTOs, the population of Southern province, and the local ecosystem.

**Table 1:** Main characteristics of the system and impact pathways for the animal health programme (AHP) and satellite project (OHI)—(+) indicates a positive or additive interaction and (−) indicates a negative or inhibitory interaction direction.

	AHP		Satellite project (OHI)
Drivers	Cattle disease	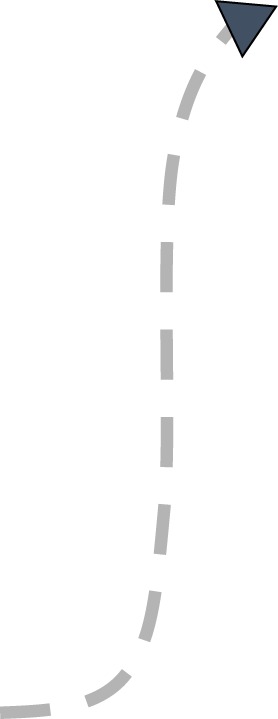	Avoid AHP unexpected outcomes
Means, measures	(+) Dipping process(traditional sectoral approach)		(+) Integrated approach(inter- and transdisciplinary, OH precursor)
1° order outcomes	(−) Cattle disease		(+) Establishment of good practices
2° order outcomes	(+) Cattle productivity and welfare (+) Farmers revenue		(+) Public health (−) Environment pollution
System outcomes	n.a.		(−) Insufficient follow-up from local institutions
Unexpected outcomes	(+) Occupational risk (+) Environmental pollution (−) Public health (chronic and acute disease)		(+) Knowledge exchange (+) Institutional learning (+) Health policy effectiveness

The theory of change for this case study is the avoidance of harm to people, plants, animals, or environment that may have arisen if an animal health driven programme of dipping cattle in OPs was implemented alone. The OHI helped to increase the sustainability of the AHP, alongside improved efficacy and efficiency for the Zambian Government looking to increase productivity in the agricultural sector. This was in addition to greater interspecies equity and better health and welfare of people, plants, animals, and the environment through the avoidance of harm and the poor welfare and financial implications such harm could have inflicted. It was also of interest to the national government and potentially international policy makers as results may be suitable for extrapolation to other countries.

#### OH-ness Evaluation, OH Index, and OH ratio

**Figure 3:**
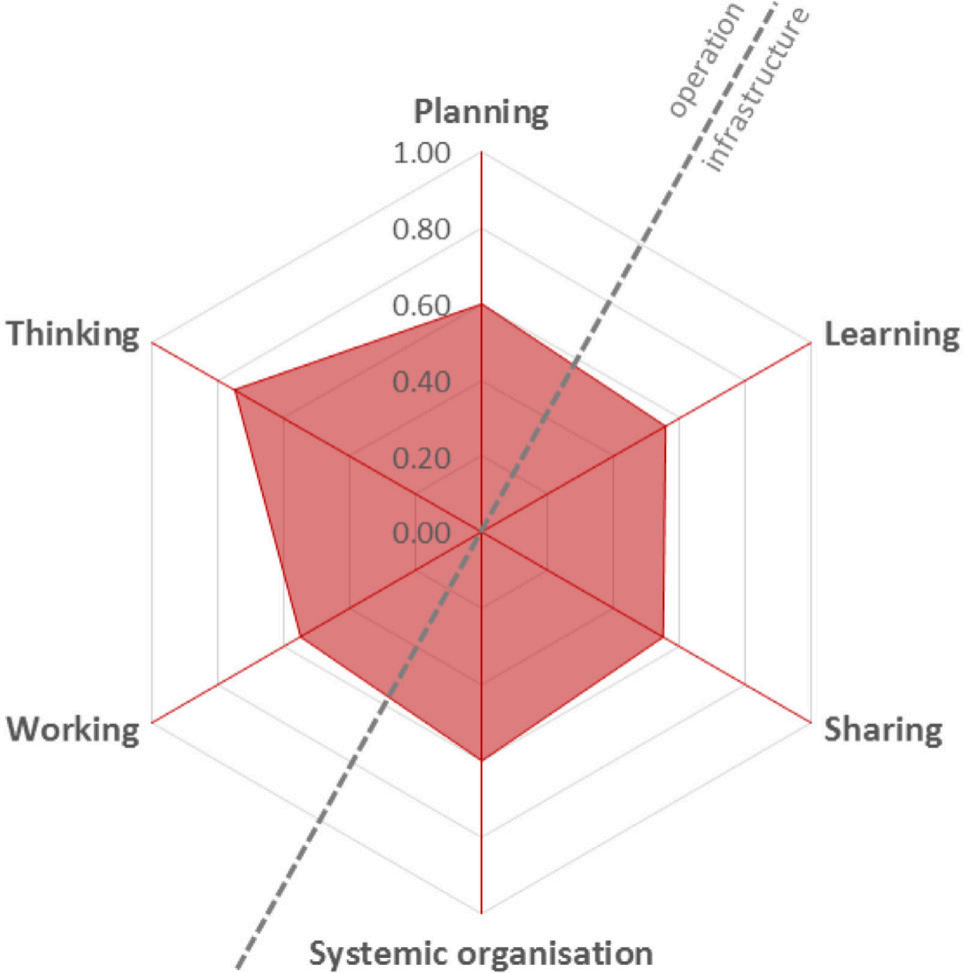
Spider diagram showing the One Health (OH) Index scores assigned for the different elements of the One Health-ness evaluation. The overall OH Index calculated from the area enclosed (shown in red) = 0.34. The hexagon is made up from the scores allocated to the OH operations (thinking = 0.63, planning = 0.60, working = 0.60) and OH infrastructures (learning = 0.55, sharing = 0.50, systematic organization = 0.60). The scores were compared to the ideal scenario (score 1.0). The One Health Ratio is calculated as a comparison of operational scores against infrastructure scores and is 1.20.

The system was scored on the six elements as set out in the NEOH framework. The spider diagram (Figure 3) demonstrates the scores for assessment of the One Health operations and infrastructure present in the initiative. The point on each spoke of the spider diagram is set from a score for OH thinking (0.63), OH planning (0.60), OH working (0.55), OH learning (0.50), OH sharing (0.55), and systemic organization (0.60) for the satellite project. Scores were allocated out of 1.0, where a high score was perfect OH criteria achieved and 0.0 was no OH criteria achieved. Scoring criteria are outlined in Tables S2a–g in Supplementary Material.

The mean score for OH thinking (0.63), as established by qualitative questionnaire review (Table S2a in Supplementary Material), was moderately high for this satellite project. The initiative had a highly integrated health approach, covering a variety of dimensions at differing scales and incorporating many perspectives. However, there was a weaker match of initiative aimed at the environment. The geographical dimension of the project targeted a global health challenge, enacted by those from multiple countries, but conducted across a short timescale. Despite the initiative impacting on whole ecosystems, these impacts were not captured. The results of this satellite project could have international economic consequences but were only utilized to inform national protocols.

An extended initiative timescale and more balanced perspective could have captured long-term impacts important for public health and other wider impacts, thereby better serving the global context of OP use to control TBD in cattle.

The mean score for OH planning (0.60) was calculated according to the match between tasks, resources and responsibilities as shown in Table S2b in Supplementary Material. Many specialist staff were utilized effectively in their areas of expertise, for example the toxicologist testing residue samples and the translation of safety advice into broadcast materials by media experts. However, there were many roles completed by those with expertise in animal health that could be more suited to human health experts or social scientists. It was noted, however, that veterinarians held a great deal of professional respect in the communities where they were operating, meaning that it was generally accepted that they had authority to deliver safety information for animal products and treatments and one veterinarian had further training in human and occupational health. A bottom-up approach to the project was utilized with stakeholders actively involved in identifying risks (interviews, etc.) and developing solutions (educational material content and discussions for lack of behavior change following delivery of education). Interaction with local medical centers was only through veterinary staff and there was a lack of follow-up so numbers of possible patients associated with OP use were unknown. However, accident-at-work reporting from the dip-tanks was monitored and captured direct exposure and inhalation toxicities. The project also aimed to build an intersectoral network.

One health working assessed the transdisciplinarity (0.60) and measured the extent to which there was cross-disciplinary working and leadership that enabled an innovative approach to the challenges in this case. The initiative was broad and flexible but suffered from an imbalance of actors. There were a larger number of those from animal health throughout but these contributors came from across multiple dimensions and scales, from local to international, with successful collaboration. Table S2c in Supplementary Material shows how stakeholders worked in an interdisciplinary way across these different dimensions to address issues impacting people, plants, animals, and the environment. There were innovative outcomes arising from the transdisciplinarity of the project through the collaboration with media and communication experts in the production of education materials for a community safety campaign. However, the satellite project may have suffered from a lack of representatives from social science and Zambian public health expertise. The initiative did, however, receive crucial input in the planning phase from an Italian doctor of public health, trained in public health promotion techniques. Despite there being warning labels for harmful exposure routes on the acaricide canisters, the identification of risk behavior and interventions to reduce it, required the involvement of a range of stakeholders and transdisciplinary working. Given the time when the satellite project was performed (late 1980s), a high level of leadership was required to both conceive of, and get accepted by both national governments, such a pioneering project. It also demonstrated adaptability by responding to results as they emerged, allowing unexpected outcomes to be captured while building a network and culture of cooperation through annual conferences.

One health learning infrastructures (0.50) scored moderately compared to the ideal adaptive and generative learning that could occur. It was more favorable at individual level with some adaptive learning, but the application of learned knowledge at individual, team, and organization level was limited (Table S2e in Supplementary Material). Feedback from end-users was readily received during several community “meetings under the tree” allowing for adaptation but was not applied during the initiative. Multimedia educational material received participation from experts and local stakeholders. There was established a yearly seminar for the provincial veterinary staff in Zambia, which also included local experts to disseminate information. Peer-to-peer learning was carried out between the Zambian animal health workers but facilitated by the Italian Institute. Institutional learning also occurred for the Italian vets working mostly for the first time in Zambia under field conditions, meaning exposure to novel challenges. Following the satellite project and AHP any continued collaboration was not formally recorded and thought to be only on a personal level. Up-dates on the present disease extent and on the methods to control Theileriosis, implemented after the termination of the Italian project, were obtained through publications, reports by FAO and Belgian Cooperation projects, and *via* personal communications with veterinary consultants who used to work in the Country. Feedback was gathered at the time of the project during face-to-face meetings, and a final handing-over project report but it has not been taken since the project ended (7).

Another moderately scored aspect of this case study was OH sharing (0.55) as outlined in Table S2d in Supplementary Material. There was good appreciation of the potential stakeholders to be involved in the project but, as previously discussed, those from human health could have played a greater role to enhance the outcomes of the project further. Data quality was variable, with some elements of the OHI being published in peer-reviewed journals (e.g., toxicology data and public health promotion activities) but in other areas there were qualitative risk assessments with no baseline case numbers for harm arising from the AHP. The full impact of the OHI was not captured adequately with only anecdotal evidence collected. However, resources were provided to allow wider sharing of risk behavior data and methods for risk mitigation both internally and externally with staff and regional animal health workers. Frequent and routine meetings were held between Zambian field staff, with provincial Zambian staff invited to a biannual meeting with national Zambian staff. There were also yearly expert missions from ISS Rome that included laboratory technicians, development cooperation, and administrative staff. Findings were used to inform a radio and television program to share findings with the public with the aim of reducing risk behavior but it is unknown if these resource intensive outputs were used after the conclusion of the project.

Systemic organization (0.60) assessed team structure, leadership and focus as outlined in Table S2f in Supplementary Material. There were multiple teams acting in the initiative with fairly clear objectives; however, some teams were of limited size and lacked formal structure for inter-team relations. The bias toward animal health sector actors was a limiting factor but despite this, the initiative was successful in utilizing an integrated system and yielded interdisciplinary outcomes.

The overall OH Index = 0.34 was calculated from the area enclosed by the points of the spider diagram (Figure 3; Table S2g in Supplementary Material). The OHI demonstrated moderate success in most areas of One Health integration. A comparison of the satellite project’s OH operations to infrastructure elements showed slightly better operational scores by managing to incorporate the input across multiple dimensions. The ratio of OH operations and infrastructure was estimated by the OHR = 1.20.

#### Economic Evaluation

Table 2 shows the breakdown of the total expenditure supported by financing organizations for the satellite project, by type of activity and relevance.

**Table 2:** Breakdown of the expenditure supported by financing organizations for the implementation of the satellite project by type of activity.

Activities	USD^a^	%
Educational and training activities (sub-total)	30,559	57.2%
– *Training activities and conferences*	*11,971*	*22.4%*
– *Radio program concept and development*	*4,448*	*8.3%*
– *Radio program production and broadcast*	*4,818*	*9.0%*
– *Production of dip-tank operator procedural manual*	*4,138*	*7.7%*
– *4-month fellowship by Istituto Italo-Africano for veterinarian counseling to the radio program production*	*3,569*	*6.7%*
– *Production of educational leaflets*	*1,615*	*3.0%*

Evaluation, monitoring and prevention of intoxication risks (sub-total)	21,041	39.4%
– *Residue testing and analysis*	*8,911*	*16.7%*
– *Assessment of occupational hazards*	*7,485*	*14.0%*
– *Provision of personal protective equipment*	*4,487*	*8.4%*
– *Provision and distribution of atropine to hospitals*	*159*	*0.3%*

Other (sub-total)	1,859	3.5%
– *Staff support at ISS Rome and WHO/FAO collaborating center*	*1,859*	*3.5%*

Total expenditure	53,460	100.0%

*^a^Original values in old Zambian Kwacha (ZMK) were converted into US Dollars: average exchange rate from January 1, 1988, to December 21, 1989, 1 USD = 11.8679 ZMK (source: www.fxtop.com, accessed on September 23, 2017)*.

Educational and training activities covered 57.2% of the total satellite project’s expenditure; the evaluation, monitoring, and prevention of intoxication risk 39.4%; and the other type of expenditure 3.5%. Total expenditure equaled about 2% of the overall budget for the AHP.

The identification of the project benefits for local communities was preceded by estimations on the acaricide use during the AHP implantation in the 90 DT stations initially targeted. According to technical data provided by project experts, in the two years of AHP and OHI operation it can be calculated that 10,035 kg of acaricide active ingredient were used, resulting in the disposal of 6,690 empty canisters and 2,430,000 l of end-of-season DT fluid containing approximately 1,020 kg of active ingredient (see Annexe A1).

The project output included production of educational activities and training materials, laboratory services, hazard assessment, and the provision of protective equipment and antidotes. This contributed to reduce the cases of OP poisoning and the environmental hazard arising from the implementation of the AHP and other similar initiatives, as well as from the general use of pesticides in the impacted area (see Annexe A2).

Annexe A3 summarizes the economic benefits of the project for the population of the beneficiary country consisting of the incomes directly and indirectly generated by employment of local staff and local purchases of goods and services, the willingness to pay of local population for the avoided cases of OP poisoning in humans and the related resource savings for the public health system, the patients and their families, and the willingness to pay of local population for the reduction of health and environmental hazards (see Annexe A3).

Due to a scarcity of data, it has not been possible to appraise the monetary value of such benefits within this study. The estimation of the incomes generated for the local population, beyond the information from the project’s technical and financial reporting, would have required other evaluations based on macroeconomic statistics of the benefited country regarding foreign trade and intermediate and final consumption of goods and services at that time (22, 23). A quantification of the prevented OP poisoning cases and an assessment of the reduced health and environmental impacts would have opened the possibility to evaluate, through the available methodologies, both: the willingness to pay of the impacted population for such benefits and the saving of resources obtained (16, 24, 25).

## Discussion

Application of this newly described framework for evaluation of integrated health projects has shown the added benefits (theory of change) from taking this pioneering approach in an earlier animal health intervention. The OHI in this case study achieved moderate success in all six evaluation themes, but failed to demonstrate long-term change or continued learning beyond its own system. As highlighted in the introductory chapter by Ruegg et al., a higher OH index does not necessarily indicate a “better” OH initiative, so it is difficult to draw comparisons of results of this case study with others at this time. However, data from this case study can and will be compared to others during further verification of the framework and in creating benchmarks in the future. Despite limited resources allocated to the completion and data collection for evaluation and the long time elapsed since project completion, it is hoped that the qualitative evaluation approach utilized here proves important in understanding short-comings and strengths of initiatives in a context-specific way.

The case study highlights clearly the wider impacts and unintended consequences that even a simple and well-established technology, such as applied for animal health here, may have on other aspects of society. The introduction of an animal health strategy in this local context (dipping and, in particular, acaricide products) did not consider relevant cultural aspects of the society such as perceptions the local population held on the level of potential risks when using an acaricide. Cultural determinants are relevant aspects of the system as they determine credence, behaviors, and material practices. This emphasizes the need to put health measures in their social context before implementation, i.e., taking a One Health approach to risk analysis and impact assessment. In this case study, AHP managers and local and national authorities were obliged to intervene due to the unexpected consequences (i.e., reuse of acaricide canisters) emerging from the AHP. It was not possible to quantitatively assess the benefits of the satellite project for the reasons already outlined. However, based on the national incidence of signs arising from the misuse of OP and related facilities, it makes sense to affirm that consequences might occur if no measures (the satellite project) were adopted. A lesson that seems still appropriate today.

The evaluation was made challenging by a lack of monitoring data to assess impacts arising from the One Health initiative. Evidence for harm arising from exposure to OPs is widely recognized in the medical literature and thus a logical model of harmful impacts was assumed based on areas where exposure was a risk. Although an integrated approach was not necessary for these harms to be predicted, the identification of risk behavior within the system would have been limited by a siloed disciplinary approach. In this case study, researchers were mostly from the animal health sector but had suitable understanding to allow detection of risks. These initial risk assessments were then supported through transdisciplinary working to develop and deliver intervention strategies.

The OHI was seemingly successful in transfer of knowledge but local stakeholder attitudes to the risks identified and communicated were “rather unmodified.” However, this was based on personal observations only. It was generally acknowledged that there was free provision of protective equipment for DTOs but its uptake was not formally recorded or successful on 100% of observations. There was also no formal recording on container reuse following modified advice, but this was sporadically observed (at least 4–5 times) suggesting a <100% success in adhering to warning messages.

The retrospective nature of the OH evaluation limited the potential impact of results. If the OH approach had been taken earlier in the intervention then it may have aided the decision-making in the planning stages, for example, taking into consderation costs or benefits arising from economical, social, and sustainability perspectives in a final decision as to the interventions viability or ethical conflicts.

The imminent health risk multiplier related to the acaricide was identified due to a mostly effective collaboration in an international, multi-disciplinary and multi-institutional team. After the implementation of the satellite project, no trace of this experience reportedly remained encoded in a structured model of institutional cooperation to ensure a similar integrated approach may be used for other problems. This underlines some relevant aspect of OH: (i) interdisciplinary and institutional cooperation is fundamental to get the advantages of OH operation; (ii) individual cooperation does not necessarily translate into institutional structures suitable for facing new problems in an effective way.

The evaluation framework according to the NEOH handbook (see text footnote 1) was applied retrospectively in this case study. This required a great deal of specific data for what was a delayed evaluation (more than 25 years after project implementation) of an informally monitored initiative. This proved challenging at times and limited the involvement of stakeholders to just a few. However, information and data were gathered for the evaluation by way of review of published and gray literature, original documentation from the initiative and semi-structured interview. Attempts to contact other stakeholders and to measure long-term impacts were unsuccessful, but the delay in evaluation in this case study is likely exceptional. It is, therefore, not suggested that this is a failing of the framework.

The AHP and the satellite project were not shaped and managed in order to provide data and information to perform current OH-ness or economic evaluation. Some data about cost were available but benefits can only be assessed in a qualitative and hypothetical way. Organizational and institutional settings adopted for the management of the satellite project are relevant for the assessment of OH-ness. They have been reconstructed through interviews with the people involved with project implementation and most of them could not be reached after approximately 25 years. Available information was compiled independently from the completion of questionnaire tools in the first instance. This allowed a more complete understanding of the initiative but this approach was time consuming and a targeted plan for information gathering to occur throughout the initiative may be desirable. The completion of the evaluation questionnaire by an external evaluator helped to reduce bias but could be vulnerable to misinterpretation on the part of the evaluator. The review of evaluation scoring criteria by multiple other evaluators, both internal and external and from differing disciplines, helped to address any bias toward the perspective of a particular evaluator. This approach proved feasible and practical for the geographically isolated evaluators in this case study. It is proposed that completion of similar evaluations could be done by external evaluators in isolation from stakeholders (thereby reducing resources required), if there existed good processes for information gathering in planning and completion of the initiative. A retrospective evaluation in this instance, therefore, proved limiting. The process of system mapping as an initial stage of evaluation lends itself well to the review of One Health initiatives. In this example, little specific data were available to map a broad system, but the mapping process allowed for assumed impact pathways (based on existing knowledge) to be used to better illustrate the context surrounding the initiative.

## Conclusion

The satellite project used a pioneering *ante-litteram* OH approach many years before the present OH definition was set and a more structured OH approach was implemented. This case study highlights the risk of implementing health interventions without consideration of the wider context and potential impacts for indirect stakeholders. The avoidance of harm and improved species equity achieved when using a One Health approach is achieved through effective transdisciplinary collaboration. In this case study, the costs of implementing such an approach were small in comparison to overall project costs, but are proposed to have had large potential impacts. This supports the implementation of One Health assessments as part of larger health programs as good value. It is recommended, however, that there be greater attention paid to determining the system, processes for monitoring and effective influence points in the planning stages to take the greatest benefit of such an approach. Routine monitoring and evaluation from a One Health perspective by utilizing the framework proposed by NEOH, could prove a valuable addition in future health strategies and as a tool for retrospective evaluation of existing policies.

## Ethics Statement

No personal information was used in this evaluation and methods used retrospective evaluation only meaning no ethical consent was sought/required for completion.

## Author Contributions

DM: general contribution in the study design; providing all background info and data concerning the AHP and the OHI activities which he carried out as (former) project consultant in Zambia; contribution in attributing the OH-ness scores; collaboration in revising, editing the manuscript; GL: primary external OH-ness and economic evaluator; SS: tertiary external OH-ness evaluator; MC: secondary external OH-ness evaluator; co-responsible of the section on “Economic evaluation”; MA: secondary external OH-ness evaluator; co-responsible of the section on “Economic evaluation”; contributor to the section “System map and Theory of Change”; all authors: drafting, editing, and reviewing the final version of the manuscript.

## Conflict of Interest Statement

The authors declare that the research was conducted in the absence of any commercial or financial relationships that could be construed as a potential conflict of interest.

## Footnotes

^1^Rüegg S, Häsler B, Zinsstag J. *A Handbook for Evaluation of One Health – Draft Version, November 2016*. (unpublished/under preparation).

## Appendix

**Annexe A1:** Technical data and estimations on acaricide use in the DT stations targeted by the satellite project.

90 DTs, mean number of operational DTS/each dipping season (average volume 16,000 l) for about 2 million cattle dips/year; DT fluid removed by dipping = 2.5 l per each cattle dip; DT replenishment after 1,000 dips = 2,500 l of DT fluid/replenishment; Total number of DT replenishments = (2 million cattle dips per year/1.000 dips) − 90 first charging = 1,910 replenishments per year; Acaricide dilution in DT fluid: 1 l acaricide/600 l of water for initial DT charging; 3 l acaricide/1,000 l of water for DT replenishments; Total acaricide utilized = (16,000 × 90 × 1/600) l + (2,500 × 1,910 × 3/1,000) l = 16,725 l per year (containing 5,017.5 kg of active principle); Acaricide canisters (5 l volume) to be disposed = 16,170/5 = 3,345canisters per year; End-of-season disposal of DT fluid by using decantation pits before dispersal on fallow land = (16,000 − 2,500) l × 90 DTs = 1,215,000 l per year (containing about 610 kg of active principle).

*Source, Ref. (3–7) and own evaluations*.

**Annexe A2:** Output description of the satellite project.

(B) Output description of the satellite project Staff trained: 95 operators of the 90 targeted DT stations, 20 Veterinary assistants, 5 laboratory assistants; Two national seminars organized for veterinarians and livestock officers; One radio program produced and broadcasted twice; Procedure manual for DT operators produced and 250 copies printed; Educational leaflets produced and 300 copies printed; Project report produced and 150 copies printed; Residues analysis in milk samples from 10 cows × 4 subsequent samplings after dipping, plus testing residues in empty canisters used to transport water and other food stuffs Assessment of occupational hazards in DT stations Personal protection equipment provided to the DTOs at the operational DTS; Atropine distributed to vet offices, health centers and hospitals in the 5 District under the AHP assistance Potential avoidance of OP poisoning cases due to implementation of the AHP and similar interventions, and more generally for pesticide use in the impacted area. Potential reduction of health and environmental risks: 12.1. Reduction of the risk of acute and chronic intoxication of DT stations’ staff caused by mishandling and bad practices in DT operations (handling of about 9,700 kg of acaricide active principle during the 2-year project period in the 90 targeted DT stations); 12.2. Reduction of the risk of acute and chronic intoxication of herders and local population caused by utilization of empty acaricide canisters for transportation and storage of water, milk, and other liquids for food and domestic uses (about 6,690 empty canisters to be disposed during the 2-year project period in the 90 targeted DT stations); 12.3. Reduction of the risks of acute and chronic intoxication of herders caused by cattle cares and operations practiced within 5 h after DT dipping; 12.4. Reduction of the risks of acute and chronic intoxication of herders and local population caused by human consumption of milk produced within 24 h after DT dipping; 12.5. Reduction of the risks of active principle dispersion in the environment caused by direct disposal of end-of-season DT fluid into fallow land without previous decantation (25,430 hl of DT fluid containing about 1,020 kg of active principle during the 2-year project period in the 90 targeted DT stations); 12.6. Improved skills of DT stations’ staff and awareness of local herders and population reduced the general risks of active principle dispersion in the environment (10,035 kg of acaricide active principle were used during the 2-year project period in the 90 targeted DT stations); 12.7. Improved skills of DT stations’ staff and awareness of local herders and population permanently reduced the risks listed from point *Error! Reference source not found*. to point *Error! Reference source not found*. for similar initiatives undertaken after the end of the project and more generally for pesticide use and handling in the impacted area.

*Source Ref. (7–9), and own elaborations*.

**Annexe A3:** Summary of the satellite project’s benefits for the population of the beneficiary country.

(i) Incomes generated by the project: – salaries and other incomes of local staff directly employed by the project; – incomes generated to local population by project’s purchases of goods and services in the territory of the beneficiary communities; – income generated to local population by project’s foreign staff personal purchases of goods and services in the territory of the beneficiary communities; – increased income derived by the reduced cattle mortality and increased productivity (ii) Willingness to pay for improvements in the health state of the population consequent to implementation of the AHP and more generally to TBD control practices and pesticide use: – potentially avoided cases of acute and chronic OP poisoning in humans related to the AHP and to similar interventions; (iii) Resource saved from potentially avoided cases of acute and chronic OP poisoning in humans: – resource saved in the public health care sector; – resource saved by patients and their households; – avoided productivity losses. (iv) Willingness to pay for reduced environmental and health hazards consequent to implementation of the AHP: – reduced risks of acute and chronic OP poisoning in humans; – reduced risks of OP dispersion in the environment; (v) Willingness to pay for reduced environmental and health hazards consequent to TBD control practices and pesticide use beyond the AHP: – reduced environmental and health hazards from general improvement of local skills and awareness regarding TBD control practices and pesticide use.

*Source: Own elaborations*.
