# Supplementary figures and images for: Control of Cattle Ticks and Tick-Borne Diseases by Acaricide in Southern Province of Zambia: A Retrospective Evaluation of Animal Health Measures According to Current One Health Concepts
Source: Front Public Health. 2018 Mar 27;6:45. doi: 10.3389/fpubh.2018.00045 (PMC5881173; doi:10.3389/fpubh.2018.00045)

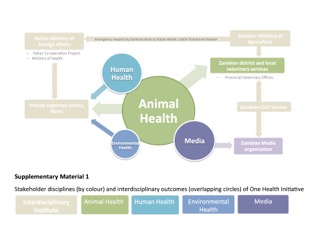

Supplement: Supplementary file 2 [file Image_1.JPEG]
